# Mitochondria: Much ado about nothing? How dangerous is reactive oxygen species production?^☆^

**DOI:** 10.1016/j.biocel.2015.01.021

**Published:** 2015-06

**Authors:** Eliška Holzerová, Holger Prokisch

**Affiliations:** a Institute of Human Genetics, Technische Universität München, Munich, Germany; b Institute of Human Genetics, Helmholtz Zentrum München, Neuherberg, Germany

**Keywords:** I, complex I, II, complex II, III, complex III, IV, complex IV, α-KGDH, α-ketoglutarate dehydrogenase, AO, alternative oxidase, cyt, cytochrome, DHODH, dihydroorotate dehydrogenase, ETF, electron transfer flavoprotein, GLRX, glutaredoxin, GPx, glutathione peroxidase, GSH GSSG, glutathione, GSR, glutathione reductase, mGPDH, mitochondrial glycerophosphate dehydrogenase, MAO, monoamine oxidase, NADH DH, external NADH dehydrogenase, NOS, nitric oxide synthase, OXPHOS, oxidative phophorylation, PDH, pyruvate dehydrogenase, PRXIII, peroxiredoxin III, ROS, reactive oxygen species, TXN2, thioredoxin 2, TXNRD2, thioredoxin reductase, Reactive oxygen species, ROS scavenging, ROS signalization

## Abstract

For more than 50 years, reactive oxygen species have been considered as harmful agents, which can attack proteins, lipids or nucleic acids. In order to deal with reactive oxygen species, there is a sophisticated system developed in mitochondria to prevent possible damage. Indeed, increased reactive oxygen species levels contribute to pathomechanisms in several human diseases, either by its impaired defense system or increased production of reactive oxygen species. However, in the last two decades, the importance of reactive oxygen species in many cellular signaling pathways has been unraveled. Homeostatic levels were shown to be necessary for correct differentiation during embryonic expansion of stem cells. Although the mechanism is still not fully understood, we cannot only regard reactive oxygen species as a toxic by-product of mitochondrial respiration anymore.

This article is part of a Directed Issue entitled: Energy Metabolism Disorders and Therapies.

**Key facts**

• Reactive oxygen species are produced in various cell compartments.
• Previously thought of as harmful agents only, they are now considered as important signaling molecules with potential therapeutic effect.

**Organelle facts**

• Mitochondria produce vital energy in the form of ATP via oxidative phosphorylation.
• Mitochondria have their own genome, called mitochondrial DNA.
• Mitochondria are responsible for most of the reactive oxygen species via oxidative phosphorylation.
• Mutations in nuclear encoded genes of mitochondrial proteins potentially result in inherited diseases, with an incidence of 1 in 10,000, most of them causing neuropathies or myopathies.
• Mitochondrial diseases of oxidative phosphorylation can be connected with increased ROS
• Mitochondria have their own ROS defense system.

## 1 Introduction

The discussion about reactive oxygen species (ROS) started around the year 1956 (Harman, 1956) with the finding that 2% of the oxygen which is used up by the respiratory chain in mitochondria can be released and transformed into a superoxide radical anion O_2_•^−^ by consuming a single electron coming from the respiratory chain. Traditionally, most of the ROS production is believed to originate from the electron transport chain in mitochondria, especially from complexes I and III. Later on, many other proteins were described as potential ROS producers, but the exact contribution from different sites is not yet fully understood. Many ROS producers arise with disruption of cell homeostasis, but, in contrast, several proteins produce ROS to restore this homeostasis. Here, we summarize sites of reactive oxygen species production and mitochondrial defense mechanisms and focus on described roles of ROS in cell signalization as a beneficial, yet often overlooked effect of ROS in cell metabolism.

## 2 Organelle function: mitochondrial sites of ROS production

**Fig. 1:**
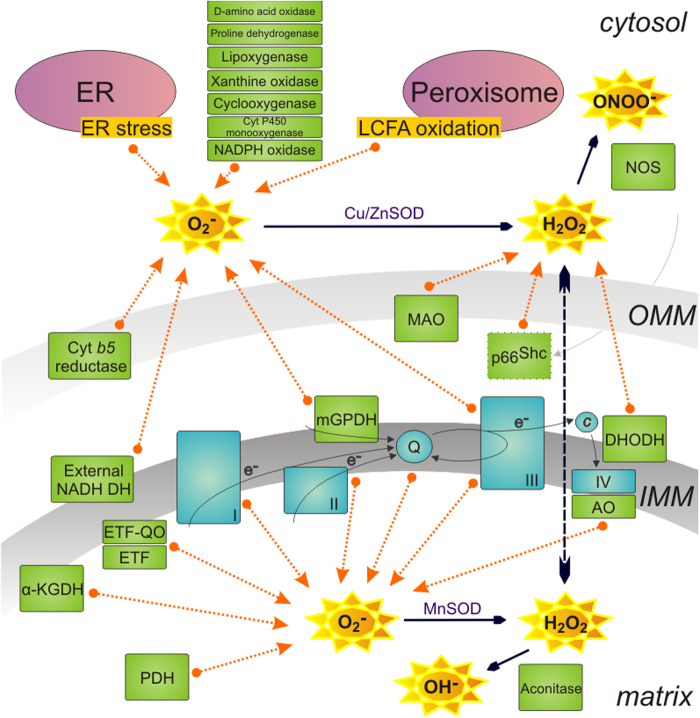
Sites of ROS production. Many different sites of ROS production exist within a cell. Most of them are located in the mitochondrial environment such as the complexes of the respiratory chain: complex I (I), complex II (II), complex III (III), or mitochondrial glycerophosphate dehydrogenase (mGPDH) next to α-ketoglutharate dehydrogenases (α-KGDH), electron transfer flavoprotein (ETF) and ETF ubiquinone oxidoreductase, pyruvate dehydrogenase (PDH), aconitase, alternative oxidase (AO), complex IV (IV), dihydroorotate dehydrogenase (DHODH), external NADH dehydrogenase (NADH DH), protein p66^Shc^, cytochrome (cyt) *b5* reductase, monoamine oxidase (MAO) and nitric oxide synthase (NOS). Other proteins or organelles can also contribute to ROS production. Respiratory chain complexes are displayed in blue, other ROS contributors in green, organelles in violet. (For interpretation of the references to color in figure legend, the reader is referred to the web version of the article.)

Mitochondria play a key role in aerobic cellular metabolism. The incomplete oxidation of oxygen to water results in superoxide production, virtually ROS. Even though it is still unclear if ROS are only harmful or beneficial, many ROS producing sites were described (Fig. 1). However, an exact contribution of each enzyme is not yet known. Complexes of the respiratory chain in mitochondria are considered as main producers, especially complex I in several sites of the enzyme (Koopman et al., 2010), complex III in subunits interacting with coenzyme Q (Raha et al., 2000; Turrens et al., 1985) and complex II under low substrate conditions (Quinlan et al., 2012) as well.

Within mitochondria, minor ROS producers can also be found. First of all, one protein, which is able to transfer electrons to the coenzyme Q pool as well as to contribute to the ROS formation, is the mitochondrial glycerolphosphate dehydrogenase (Drahota et al., 2002). It is located in the inner membrane facing the intermembrane space. Another significant contribution to ROS production occurs during fatty acid oxidation due to electron transfer flavoprotein (ETF) that accepts electrons from different dehydrogenases and transfers them through its membrane partner ETF ubiquinone oxidoreductase to the coenzyme Q pool in the inner membrane (Ruzicka and Beinert, 1977). Next, there is a multisubunit pyruvate dehydrogenase complex (Starkov et al., 2004) and a structurally similar membrane bound enzyme complex of α-ketoglutarate dehydrogenase (α-KGDH) which has been proposed as a source of superoxide and hydrogen peroxide under low availability of NAD^+^, the natural electron acceptor of α-KGDH (Starkov et al., 2004). Many of the aforementioned proteins contain flavin in their active site, which is directly interacting with electrons and plays a possible role in the electron leakage.

Aconitase, an enzyme in the mitochondrial matrix, is able to transform hydrogen peroxide into hydroxyl radicals during a Fenton reaction with its iron–sulphur cluster. Aconitase, though, is easily inhibited by the presence of superoxide (Vasquez-Vivar et al., 2000). The function of many proteins is changed upon oxidative stress in cells. Redox disbalance and subsequent oxidation of, for example, protein p66^Shc^, which plays an important role in the regulation of apoptosis, translocates it into the intermembrane space to produce H_2_O_2_ (Pelicci, 2005).

There are several other proteins contributing to mitochondrial ROS production either as a superoxide or as a hydrogen peroxide, most of them non-mammalian: external NADH dehydrogenase (Fang and Beattie, 2003b) or alternative oxidase (Fang and Beattie, 2003a), proline dehydrogenase (White et al., 2007), cytochrome b5 reductase (Whatley et al., 1998), monoamine oxidase (Koopman et al., 2010) and dihydroorotate dehydrogenase (Forman and Kennedy, 1976) as well as potentially complex IV (Koopman et al., 2010).

Next to the mitochondrial ROS production, numerous contributing proteins were identified in the cytoplasm as well. Most important is the family of NADPH oxidases, proteins crucial for host defense and killing of microorganisms during phagocytosis due to increased ROS production (Hampton et al., 1998). Special conditions lead to increased ROS production in other organells too: upon stress, such as the unfolded protein response in the endoplasmatic reticulum and as part of the long-chain fatty acid oxidation in peroxisomes (Holmstrom and Finkel, 2014). In the cytoplasm or within organelles we find producers like lipoxygenase, xanthine oxidase, cyclooxygenase, cytochrome P450 monoxygenase as well as d-amino acid oxidase (Holmstrom and Finkel, 2014). Nitric oxide synthase is a protein responsible for the formation of reactive nitrogen species (Koopman et al., 2010) in the cell cytoplasm. An overview of proteins so far reported as possible ROS producers within or outside mitochondria is displayed in Fig. 1.

## 3 Cell physiology: ROS scavenging and signalization

**Fig. 2:**
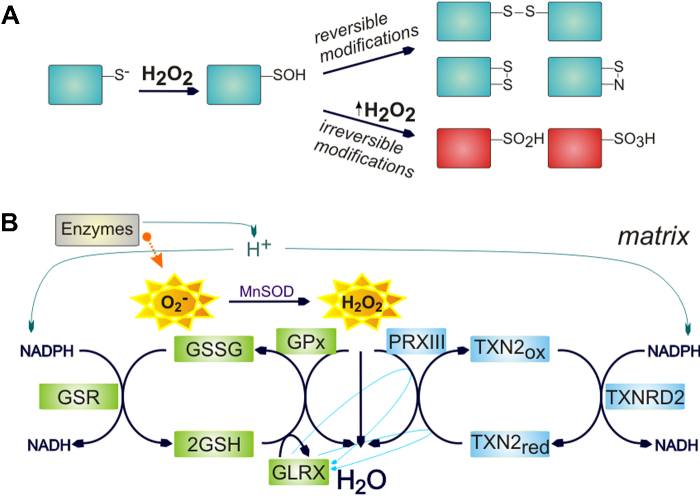
Principle of ROS signalization and ROS scavenging mechanism. (A) Redox signalization causes reversible or irreversible modification in redox sensitive proteins in thiol molecules of cysteine residues. (B) ROS are produced within oxidative phophorylation (OXPHOS) or by other proteins. Reversible redox modifications are restored due to proteins or molecules of the ROS scavenging systems of thioredoxin and glutathione. Within mitochondria, hydrogen peroxide is sensed by peroxiredoxin III (PRXIII) and oxidation of PRXIII is reduced by thioredoxin 2 (TXN2) with help of thioredoxin reductase 2 (TXNRD2). In the glutathione pathway, glutathione peroxidase (GPx) reduces H_2_O_2_ and it is subsequently sensed by glutathione (GSH) molecule, which forms dimers (GSSG). The GSH dimers are reduced by the glutathione reductase (GSR) or by a molecule of glutaredoxin (GLRX).

Thiol groups within redox-sensitive proteins are most important not only for ROS scavenging but also for ROS signaling. Cysteine residues, especially with low p*K*_a_ and a thiolate anion (—S^−^) at a physiological pH, are the most prominent responders to redox changes, but methionines, tryptophans and tyrosines are prone as well. Besides, iron–sulphur clusters containing proteins provide this sulphur from their cysteine residues (e.g. aconitase). An attack of the peroxide bond leads to the formation of reversible sulphenic acid (—SOH), which is reactive and can easily be transformed into a disulfide bridge (Fig. 2A). These changes in thiol groups are reverted by glutathione or thioredoxin systems (Fig. 2B) with the help of the peroxiredoxin family of enzymes. However, upon ongoing exposure to hydrogen peroxide in the microenvironment, sulphenic groups can be further oxidized into irreversible sulphinic (—SO_2_H) or sulphonic (—SO_3_H) acids (Koopman et al., 2010).

The ROS scavenging mechanism occurs mainly in mitochondria, but analogous proteins exist in the cytosol. Specifically in peroxisomes, organelles producing ROS for their functioning, we find catalase, an enzyme capable of decomposing hydrogen peroxide to water and oxygen, whereas mitochondria have very low levels of this enzyme. The same removal of hydrogen peroxide is also used in mitochondria by peroxiredoxin and glutathione peroxidase (Fig. 2B), which are oxidized in return. Proteins of the peroxiredoxin family form dimers upon oxidation, which are oxidized by thioredoxins. Oxidized thioredoxin is then reduced by thioredoxin reductase in the presence of NADPH. In the other part of the pathway, oxidized glutathione peroxidase is reduced by a molecule of glutathione which forms dimers that are restored by the glutathione reductase. Glutathione is part of the cellular nonenzymatic antioxidant system also including vitamines C and E, carotenoids and flavonoids (Koopman et al., 2010). Reduction of glutathione peroxidase can also be performed by glutaredoxin, a molecule providing a possible crosstalk with the thioredoxin pathway, specifically with peroxiredoxin and thioredoxin (Hanschmann et al., 2010). On the other hand, thioredoxin reductase can directly affect peroxiredoxin as well as glutaredoxin and glutathione is able to reduce thioredoxin directly (Casagrande et al., 2002).

Due to indirect inhibition of proteins by oxidation of their thiol group, some of the signaling pathways become unblocked and therefore active. This is specifically true for the inactivation of protein phosphatases by H_2_O_2_, thereby increasing the level of protein phosphorylation (Meng et al., 2002). In contrast, redox dependent inactivation of protein tyrosine phosphatases may be specific and reversible (Denu and Tanner, 1998). ROS can also directly affect kinase signaling, for example a receptor tyrosine kinase (Truong and Carroll, 2013). A second example for the direct role of ROS in signal transduction was observed with increased tyrosine phosporylation occurring after growth factor stimulation preceded by a burst of ROS generation (Bae et al., 1997; Sundaresan et al., 1995). As previously mentioned, ROS attack DNA, but their signalization affects transcription factors, e.g. bacterial OxyR, as well. This redox sensitive protein regulates the antioxidant stress response. Oxidation of cytosine residues in OxyR creates an intramolecular disulphide bond locking the transcription factor in its active configuration (Lee et al., 2004; Xanthoudakis and Curran, 1992). Many other pathways and proteins such as actin polymerization, activity of the calcium/calmodulin-dependent kinase, DNA binding of transcription factors activator protein 1 or forkhead box protein O, pathways of Kelch-like ECHS-associated protein 1 and nuclear factor erythroid 2-related factor 2, essential autophagy protein ATG4 are all connected with ROS production (Holmstrom and Finkel, 2014). Additionally, various roles were described in circadian rhythms, innate immunity, metabolic regulations gut homeostasis, stem cell biology, the pathogenesis of cancer and why and how we are aging (Holmstrom and Finkel, 2014). In many of these mechanisms, mitophagy caused by increased ROS production serves as a quality control system. Based on several observations, a principle of redox signaling reveals:

Mutation in gene/Specific conditions → ↑ ROS → ↑ ROS signaling → higher compensation → (PARTIAL) recovery of normal status

## 4 Organelle pathology: impairment of ROS scavenging system

A number of mutations has been described in genes coding for respiratory chain complexes, some of them increasing ROS production, some of them having no effect. To date, no significant positive effects of antioxidant treatments were observed within properly performed clinical trials (Pfeffer et al., 2013). On one hand decreasing ROS amount in patients had no effect on disease improvement. On the other hand no increased ROS production was observed in the mutator mouse model, which is accumulating mutations in mitochondrial DNA over time (Bratic and Larsson, 2013), nor in mouse model with mutations in NDUFS4 subunit of complex I (Chouchani et al., 2014). The subunits of the respiratory chain complexes are impaired by the mutations, yet in these cases ROS are not part of the pathomechanism. In case of natural ROS increase, reactive radicals are removed as previously described by compensatory mechanisms. Nevertheless, several pathologies concerning the scavenging mechanism have been revealed in the past few years. Recently, Familial glucocorticoid deficiency caused by mutations in the mitochondrial antioxidant thioredoxin reductase (TXNRD2) has been described (Prasad et al., 2014). Several living patients were reported in this study. However, in vitro experiments demonstrated that the glutathione system is unable to fully compensate for the TXNRD2 deficiency leading to increased mitochondrial superoxide production. Moreover, a mouse knock-out model showed to be embryonic lethal (Conrad et al., 2004). This is also the case for a thioredoxin 2 mouse model, where only heterozygous mice are surviving (Nonn et al., 2003). A frameshift mutation in human glutaredoxin 2 causing hearing loss was also reported, yet unfortunately, impaired ROS production was not investigated (Imtiaz et al., 2014). A state characterized by low level of catalase in the cell environment is called acatalasemia. This is not directly causing a disease, but it can contribute to increased risk of developing other disorders. Understanding the whole mechanism of ROS scavenging can help to clarify specific problems and potential therapies in such condition. Concerning all so far reported mutations, we can conclude that this system is quite robust and even with some missing pieces, it is able to compensate or just skip lacking part via a functioning crosstalk.

## 5 Future outlook

Although redox signalization seems to be less specific than other types of signalization, mechanisms like controlled production, cellular localization, time and exposure of cysteine residues play an important role. Also, rapid and reversible changes of protein function create this extraordinary signalization. Special attention should be applied to the specific level of ROS occurring in the cell environment, making it one of its greatest aspects. Principles can be similar in many cases, revealing potential selective therapeutic directions as well. Therefore, it is essential to identify definite targets and their function. It can be expected that genome sequencing of patients will discover more mutations in the defense system. This will contribute to our understanding of the system. Particular potential lies in H_2_O_2_ which exists naturally in 10 nm intracellular concentration and, as it is not charged, is not as reactive as other radicals and can pass through membranes.

Many diverse details remain to be elucidated and many questions to be answered, but we already have several hints. ROS increase during both inhibited and reduced amount of substrates, hypoxia and hyperoxia or endogenous and exogenous stress. Hence, diverse mechanisms must exist to differentiate contrasting problems and come to different solutions. Moreover, the origin of ROS was not properly investigated. Do we need ROS because they protected the original prokaryotic cell to survive in a dangerous environment (as current NAPDH oxidases) or did they develop later on purpose? How do we influence the level of ROS production? How can we gain anything out of that? For this and many other reasons, a great field of redox biology is opened for further research.
